# Improper Primate Release Fuels Interspecific Mating: Cases of Two Mixed‐Species Groups in Bangladesh

**DOI:** 10.1002/ece3.72279

**Published:** 2025-10-16

**Authors:** Tanvir Ahmed, Atikul Islam Mithu, Auritro Sattar, Rasel Debbarma, Harish Debbarma, Sajid Hassan Prangon, Md. Ashiqun Nabi Litu, Safayat Hossain, Abdullah As Sadique, Fa‐Tu‐Zo Khaleque Mila, Sabit Hasan, Shimul Nath, Christian Roos

**Affiliations:** ^1^ Primate Genetics Laboratory, German Primate Center Leibniz Institute for Primate Research Göttingen Germany; ^2^ Faculty of Biology and Psychology, Georg‐August University School of Science University of Göttingen Göttingen Germany; ^3^ Phayre's Langur Conservation Initiative in Bangladesh Dhaka Bangladesh; ^4^ Department of Zoology, Faculty of Life and Earth Science Jagannath University Dhaka Bangladesh; ^5^ Department of Environmental Sciences Jahangirnagar University Savar Bangladesh; ^6^ Tipra Para, Satchari, Habigonj Bangladesh; ^7^ Creative Conservation Alliance Dhaka Bangladesh; ^8^ Wildlife Crime Control Unit, Bangladesh Forest Department Dhaka Bangladesh; ^9^ Wildlife and Nature Conservation Circle, Bangladesh Forest Department Dhaka Bangladesh; ^10^ Isabela Foundation Dhaka Bangladesh; ^11^ Gene Bank of Primates German Primate Center, Leibniz Institute for Primate Research Göttingen Germany

**Keywords:** exotic species, hybridization, mismanagement, polyspecific associations, translocation, wildlife trafficking

## Abstract

Wildlife trafficking has escalated in recent years, leading to a rise in animal rescues every year. In Bangladesh, increasing numbers of primates are being rescued from illegal trades and private collections. Often, these confiscated individuals are released into the wild without proper assessment and rigorous planning. The survivability of these animals, as well as the potential negative impacts on both the released individual and recipient ecosystems, has not been systematically evaluated. In this study, we document two cases of misguided primate releases outside their natural ranges with potentially significant consequences. At two sites, two released individuals, an Assamese macaque ( *Macaca assamensis* ) and a capped langur ( *Trachypithecus pileatus* ), integrated into groups of native primates and engaged in interspecific mating. Community interviews further indicated another improper release of an Assamese macaque at another site. These incidents likely resulted from species misidentification and raise serious concerns regarding mixed‐species associations and potential hybridization between native and non‐native primates. Collectively, our findings underscore the need for rigorous species‐level verification prior to releases, adherence to IUCN translocation guidelines, and development of national protocols to mitigate human‐mediated hybridization risks and to safeguard native biodiversity.

## Graphical abstract

In Bangladesh, confiscated animals are often released without proper assessment, risking harm to native populations. We reported locally non‐native primates forming mixed‐species groups with native primates and engaged in interspecific matings, raising concerns about hybridization risks. Our findings underscore the need for rigorous species‐level verification prior to releases, adherence to IUCN translocation guidelines, and development of national protocols to mitigate human‐mediated hybridization risks and to safeguard native biodiversity.

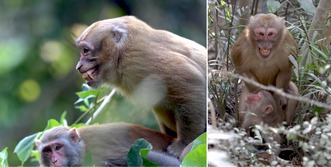

## 1 Introduction

Wildlife trafficking is a multibillion‐dollar global problem that captures countless animals annually, leading to a significant biodiversity loss (Garber et al. 2024). Non‐human primates (hereafter, primates) constitute a substantial proportion of the trafficked animals that are accelerating population declines in many threatened species (Estrada et al. 2017; Garber et al. 2024). In Bangladesh, primate trafficking appears to have escalated in recent years despite legal protections (Maria et al. 2025). Analysis of media reports revealed that the globally vulnerable capped langur ( *Trachypithecus pileatus* ) is the most affected species by illegal trading in Bangladesh, followed by the endangered Bengal slow loris ( *Nycticebus bengalensis* ), Phayre's langur ( *T. phayrei* ), and Western hoolock gibbon ( *Hoolock hoolock* ) (Maria et al. 2025). While macaques are likely to be less affected, wild‐caught Assamese macaques ( *Macaca assamensis* ), rhesus macaques ( *M. mulatta* ), and Northern pig‐tailed macaques ( *M. leonina* ) are traded locally to be used as performing monkeys and for showcasing in mini‐zoos or to be kept as pets (Akhtar et al. 2014; IUCN Bangladesh 2015). Meanwhile, the stump‐tailed macaque ( *M. arctoides* ) and long‐tailed macaque ( *M. fascicularis* ) are considered extinct in the wild or missing species, lacking any recent confirmed sighting in the country (Chetry et al. 2020; Hansen et al. 2022). Although there is no published information on the trafficking of Bengal‐sacred langurs ( *Semnopithecus entellus* ), they were found in many illegal mini‐zoos (WCCU 2025). However, the true extent of illegal primate trafficking in Bangladesh remains largely unknown.

In response to this burgeoning illegal trade, the Bangladesh Forest Department has actively seized numerous live primates from illegal trade in collaboration with national law enforcement agencies and local community networks (Akash 2025). The Wildlife Crime Control Unit (WCCU) of the forest department conducted further enforcement operations targeting illegal mini‐zoos and private collections to combat both domestic and transboundary wildlife trafficking (TBS Report 2025; WCCU 2025). As a result of these efforts, many illegally kept primates have also been confiscated. Ideally, these confiscated primates should return to their original populations or be translocated to suitable habitats within their native range (Oklander et al. 2020). But in cases of confiscated animals, the origin is usually unclear. Thus, the animals are subsequently relocated to various forest sites across Bangladesh, including Madhupur National Park in the north‐central region, Lawachara and Satchari National Parks in the northeast, and Chunati and Hajarikhil Wildlife Sanctuaries in the southeast, while some individuals have been placed in rescue centers, safari parks, or national zoos (WCCU 2025).

Despite these interventions, the release of confiscated primates in Bangladesh has often been ad hoc or uninformed, and the survivability and adaptation of released individuals remain unassessed. Moreover, the potential negative impacts of unregulated releases on both translocated individuals and recipient ecosystems, including the risk of interspecific mating and hybridization between native and non‐native primates, have not been systematically evaluated. To address this knowledge gap and inform better management strategies, we investigated two cases of improper primate release in Bangladesh that have potentially alarming consequences.

## 2 Methods

### 2.1 Study Areas

**FIGURE 1:**
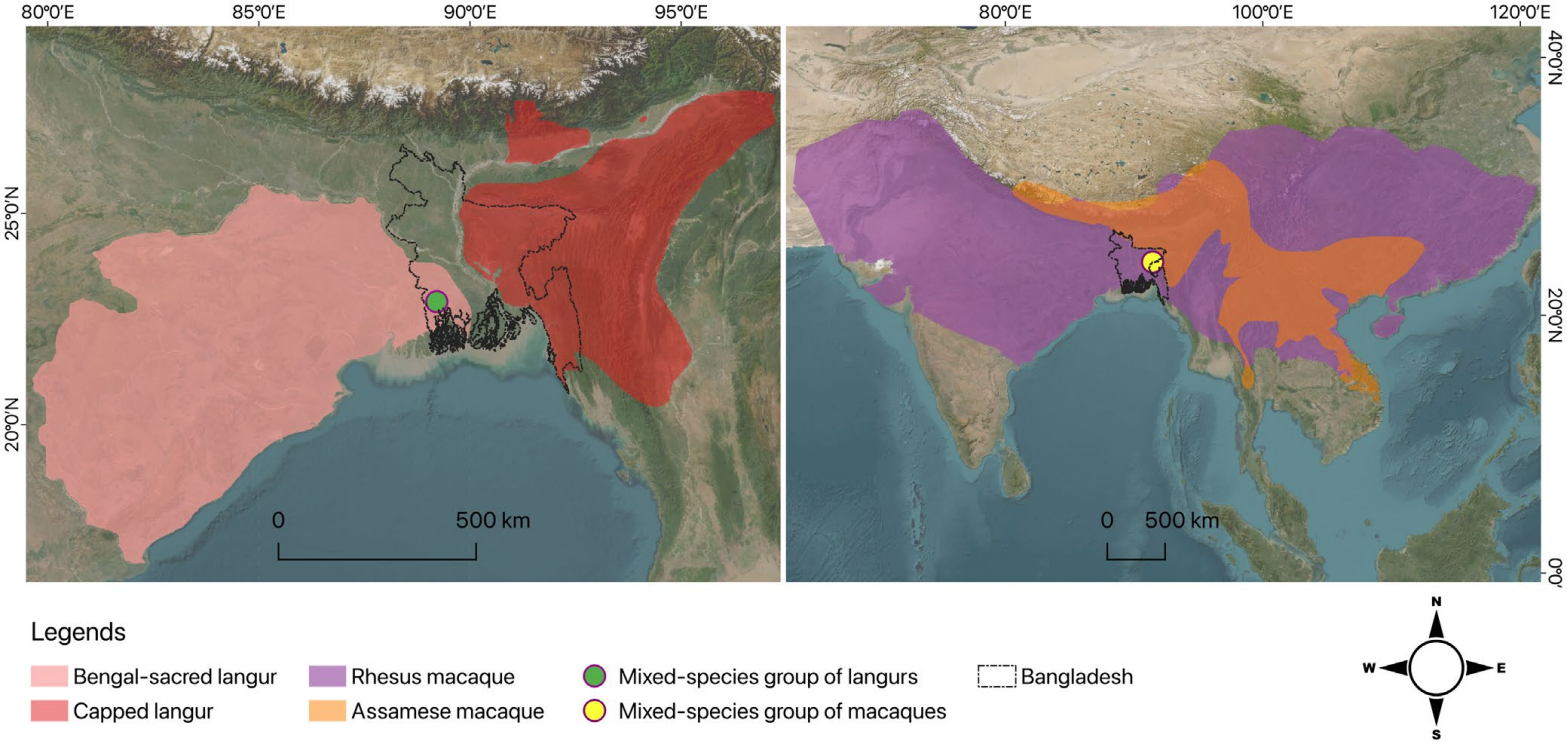
Global distribution of study species and geographic positions of the mixed‐species groups where interspecific mating occurred. The source of the distribution shapefiles is https://www.iucnredlist.org/.

We surveyed primates in 28 forest sites in Bangladesh between 2023 and 2025. The two sites, Satchari National Park and Keshabpur town, were chosen for this study due to unexpected occurrences of two primate species outside of their known distribution range (Figure 1). Satchari National Park is a 2.43‐km^2^ mixed‐evergreen forest surrounded by industrial tea plantations in the Sylhet Hills bio‐ecological zone in Northeast Bangladesh (Ahmed and Naher 2021). The forest is home to several globally threatened animals, including six native primate species: the Western hoolock gibbon, Phayre's langur, capped langur, Northern pig‐tailed macaque, rhesus macaque, and Bengal slow loris (Ahmed and Naher 2021). On the other hand, Keshabpur is a small town in the southwestern region with mosaic homestead vegetation that supports most of the Bengal‐sacred langur populations in the country but no other primate species (IUCN Bangladesh 2015).

### 2.2 Study Species

This study presents cases related to four primate species: rhesus macaque, Assamese macaque, capped langur, and Bengal‐sacred langur. Among these, the rhesus macaque is the most widely distributed and adapted non‐human primate species globally, found across all major habitat types in Bangladesh (IUCN Bangladesh 2015). Rhesus macaques are sympatric with Assamese macaques, and the latter have scattered populations restricted to eastern mixed‐evergreen and hill forests in the country (Hasan et al. 2022). Both macaque species are similar in appearance but differ in body mass, facial pattern, and coloration (Hasan et al. 2022). Assamese macaques in Bangladesh have a brown to dark brown coat color, white to brown underside, and a longer, thinner tail, while rhesus macaques have a yellowish gray body, gray shoulders, and a shorter, thicker tail (Anandam et al. 2013).

Similar to the macaques, the capped langur also has a wide global distribution range, and in Bangladesh, it is found in north‐central deciduous forests and eastern mixed evergreen and hill forests (IUCN Bangladesh 2015; Ahmed et al. 2025). The westernmost distribution limit for capped langurs is the Brahmaputra–Jamuna–Padma–Meghna River system in Bangladesh, which is eventually the easternmost distribution limit for the Bengal‐sacred langurs (Roos et al. 2014; IUCN Bangladesh 2015). Both langurs have black faces, but they significantly differ in other phenotypic colorations (Anandam et al. 2013). The capped langur has a black to light gray crown with prominent ‘horn’ like tufts, contrasting gray‐to‐brown coats and white‐to‐red orange underside, and the coats in Bengal‐sacred langurs vary from gray to yellowish gray with a white underside and a rounded gray crown (Anandam et al. 2013; Choudhury 2014). However, both macaque and langur species show considerable pelage variations all over their distribution range (Anandam et al. 2013; Choudhury 2014).

### 2.3 Data Collection

Between October 2023 and September 2025, we conducted surveys on the distribution, populations, and threats of the primates in Bangladesh, with a special focus on the globally threatened langurs. In addition to collecting population data within their known distribution range (hereafter, “native”; IUCN Bangladesh 2015), whenever a primate was found outside its known range (hereafter, “non‐native”; IUCN Bangladesh 2015), we noted the species name, age‐sex category, location name, and GPS coordinates. Opportunistically, we recorded interspecific mating behavior of these non‐native with native species following the Ad libitum observation method (Altmann 1974; Hasan et al. 2024). We also employed four trained research assistants (RD, HD, SHP, MANL) to monitor the individuals of non‐native species at least once a month to assess whether non‐native species formed mixed‐species groups with any native primates (Ahmed et al. 2025). We used Vortex 8 × 42 binoculars to observe the animals and digital cameras to capture photographic evidence of the particular behaviors, such as grooming and mating events, between native and non‐native species. Furthermore, we conducted informal interviews with regional forest officials and local eco‐guides to investigate the origin of non‐native individuals.

## 3 Results

During separate surveys, we recorded two individuals of non‐native primate species at different sites. These individuals formed mixed‐species groups with native primates, within which we observed nine interspecific mating events. However, no potential hybrid offspring were detected.

### 3.1 Case 1: Assamese Macaque in Satchari National Park, Habiganj

**FIGURE 2:**
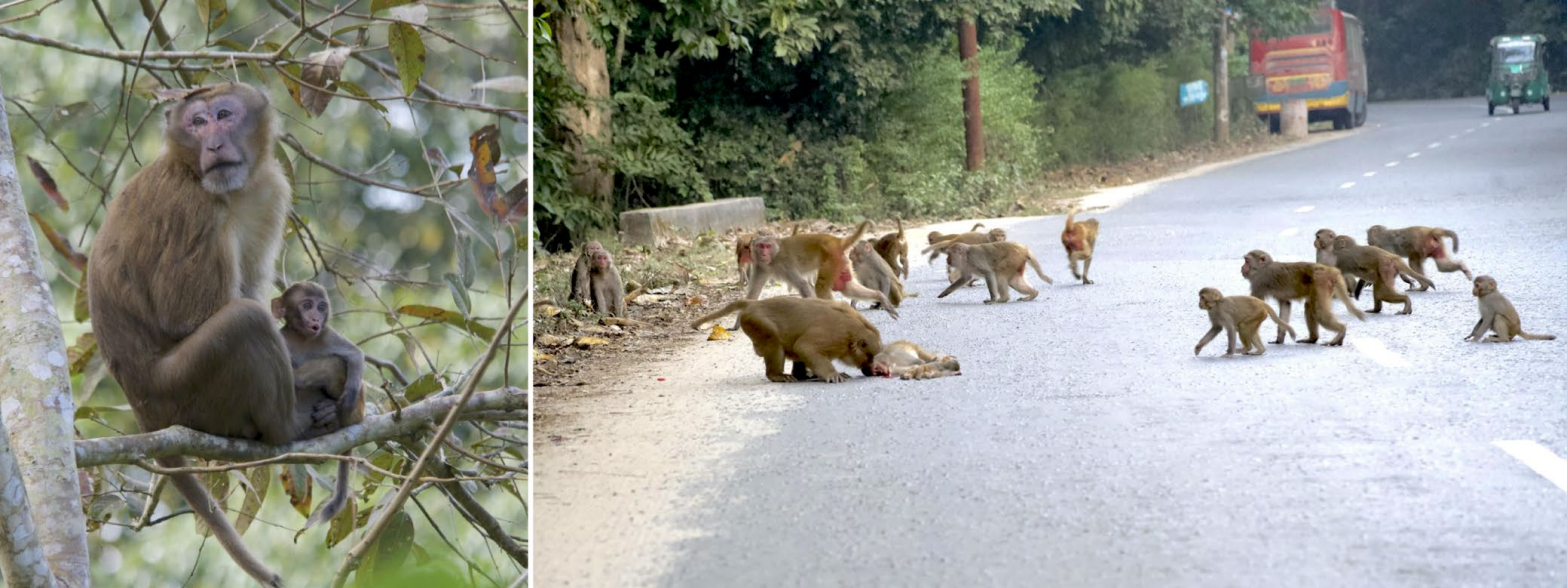
The Assamese macaque holding an infant (left) and checking rhesus macaque carcasses (right) in Satchari National Park, Northeast Bangladesh.

On July 5, 2021, we recorded an adult male Assamese macaque with distinct facial markings along the animal‐watching tower road at Satchari National Park. This non‐native individual formed a mixed‐species group with 38 rhesus macaques and acted as the most dominant member. On one occasion, the Assamese macaque was observed holding a rhesus macaque infant while the presumed mother fed nearby, but did not approach. The male later carried the infant about 30 m along the road into dense vegetation. On December 7, 2024, two rhesus macaques from the group (an infant and its presumed mother) were killed in vehicle collisions; the Assamese macaque investigated the carcasses first like a group leader, while other group members observed from 1 to 2 m away (Figure 2).

**FIGURE 3:**
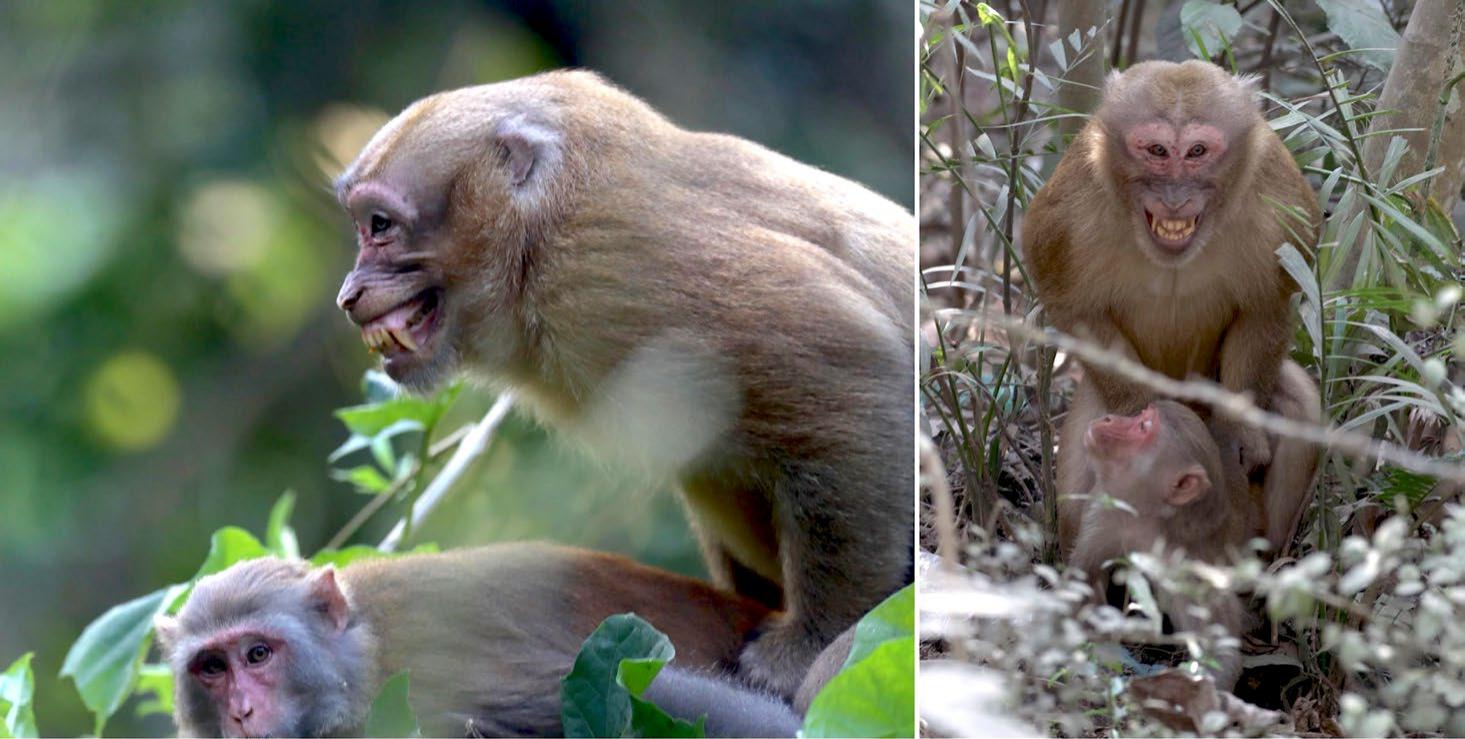
The non‐native Assamese macaque mating with two individuals of native rhesus macaques in Satchari National Park.

During the study period, we recorded eight mating events between the Assamese macaque and female rhesus macaques (Figure 3; File S1). Copulations were ventro‐dorsal, lasting 4–21 s (mean 11 s). The male initiated mating and displayed dominance. In one case, a female forcibly removed the male's genitalia, which resulted in external semen discharge. We observed the male ingesting two small portions of thick smegma from its genitalia.

### 3.2 Case 2: Capped langur in Keshabpur, Jessore

On June 12, 2024, we first recorded a sub‐adult male capped langur, a non‐native species in the region, with a group of 14 Bengal‐sacred langurs at Keshabpur Upazila Health Complex. During this observation, the capped langur occasionally held a juvenile, while an adult male Bengal‐sacred langur briefly chased him. Subsequently, over the next 4 months, the capped langur remained with the group and occasionally foraged alongside an adult female Bengal‐sacred langur.

**FIGURE 4:**
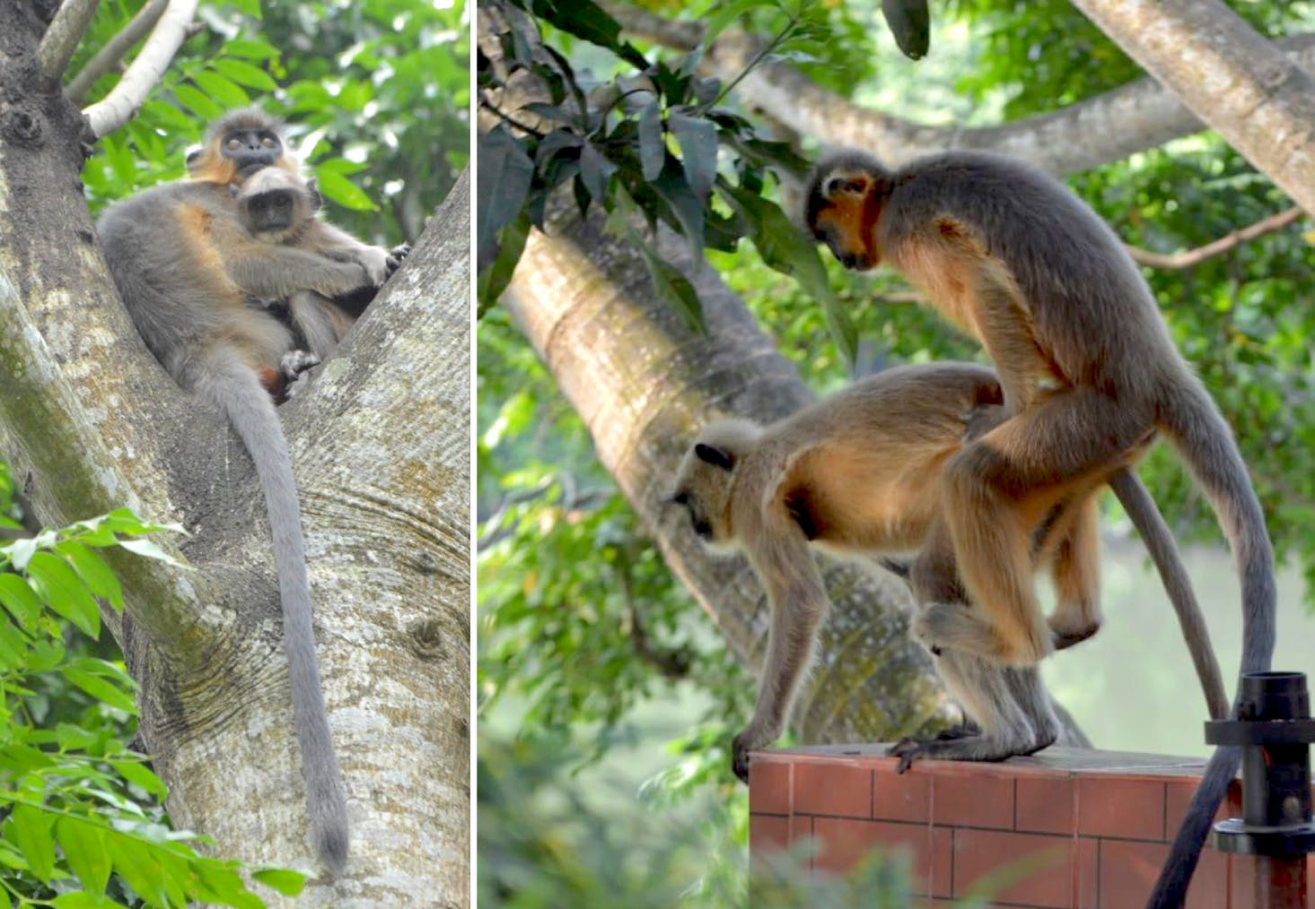
An adult male capped langur holding an infant (left) and mating with an adult female Bengal‐sacred langur (right) at the Keshabpur Upazila Health Complex, Jessore.

Later on, we recorded two copulations between these native and non‐native langurs. On November 24, 2024, after 50 s of grooming, the male capped langur copulated for 16 s with two thrusting bouts and rested 45 s with an adult female Bengal‐sacred langur (Figure 4). Similarly, on December 25, 2024, following 115 s of grooming and genital rubbing, the male copulated for 19 s with two thrusting bouts. However, it was unclear whether the female was the same in both events.

Informal interviews with local eco‐guides, researchers, and regional forest officials did not provide precise information on the timing or parties responsible for translocating non‐native primates at the two sites. Nevertheless, we obtained preliminary information on a potential third case: an injured adult male Assamese macaque, rescued from Moulvibazar, treated at Jankichora Wildlife Rescue Center, and subsequently released into Lawachara National Park. Post‐release monitoring was unknown, and we did not encounter this individual during our surveys.

## 4 Discussion

We documented two confirmed and one potential case of improper primate release in Bangladesh, involving two Assamese macaques and one capped langur outside their known ranges. In both confirmed cases, the non‐native primates formed stable mixed‐species groups with native primates and engaged in interspecific mating. While the Assamese macaque in Lawachara National Park appears deliberately released, the reasons for the other introductions are unknown.

We propose three potential causes for these improper releases of non‐native primates: (1) species misidentification, (2) accidental transportation via hitchhiking on fruit‐transport vehicles, and (3) deliberate release from captivity or personal collection. First, the rhesus and Assamese macaques, as well as capped and Bengal‐sacred langurs, are similar in general appearance (Anandam et al. 2013), which may lead to species misidentification for non‐expert personnel. Second, accidental long‐distance transport via fruit‐carrying vehicles cannot be ruled out, given that the nearest Assamese macaque populations are located approximately 70 km from Satchari (Jhimai Tea Estate, Bangladesh, Hasan et al. 2022; Udaipur–Amarpur main Road, India, Choudhury 2018) and capped langurs approximately 200 km from Keshabpur (Madhupur National Park, IUCN Bangladesh 2015). Third, deliberate release by private holders or unauthorized parties is also possible. Among these, species misidentification appears to be the most plausible explanation, underscoring the need for proper species identification training among the regional frontline staff of the Forest Department.

Our study shows that improper releases facilitate stable mixed‐species associations and interspecific mating between native and non‐native primates, although no hybrid offspring were detected. Similarly, the reported association between Hatinh langurs ( *T. hatinhensis* ) and red‐shanked doucs ( *Pygathrix nemaeus* ) in Vietnam was likely derived from released individuals from illegal trafficking (Nguyen et al. 2024), while in Brazil, uninformed releases of pet marmosets ( *Callithrix jacchus* and *C. penicillata* ) resulted in hybridization with heterospecific populations (Malukiewicz 2019). In such contexts, some of the potential consequences may include non‐native individuals outcompeting natives for resources or mates, causing stress, aggression, or reproductive failure and facilitating disease transmission (IUCN/SSC 2013; Warne and Chaber 2023).

In addition to mixed‐species interactions, copulatory thrust durations in our study (4–21 s, File S1) indicate variation in reproductive behavior that may influence hybridization risk, with longer durations potentially increasing insemination success (Dixson 2012; Birkhead and Pizzari 2002; Parker 1970). Some individuals may be more likely to successfully mate with non‐native or released primates, providing a behavioral pathway for hybridization. Historical and field evidence support this risk, with hybridization reported between capped and Bengal‐sacred langurs in captivity (Gray 1972) and between Assamese and rhesus macaques in the wild (Menon 2014). Over time, such unnatural associations could erode reproductive barriers through frequent hybridization events between native and non‐native primates, compromise genetic integrity, and alter natural selection, highlighting the implications of anthropogenic translocations (Theodoropoulos et al. 2025).

To reduce these risks, we strongly recommend the immediate removal of non‐native individuals from Satchari and Keshabpur and subsequently releasing them into suitable habitats within their natural ranges. Immediate priorities should include expert‐led species verification and careful selection of release sites. Currently, Bangladesh lacks a national protocol for the management and release of confiscated animals. Hence, developing a national framework focused on primates, in accordance with the IUCN Guidelines for Reintroductions and Other Conservation Translocations (IUCN/SSC 2013), will be a critical step forward. Development of such protocols is within the scope of the country's Wildlife (Conservation and Security) Act, 2012, and aligns with the broader biodiversity conservation goals of the Bangladesh Biodiversity Act, 2017.

## 5 Conclusion

We presented three cases of improper primate releases outside of their known distribution range, which have resulted in mixed‐species group formation and increased risks of hybridization between native and non‐native primates. These incidents underscore the urgent need for improved management strategies for confiscated primates, development, and stricter enforcement of wildlife release protocols to protect native primate populations and maintain ecological integrity.

## Author Contributions

**Tanvir Ahmed:** conceptualization (lead), data curation (lead), formal analysis (lead), funding acquisition (lead), investigation (lead), methodology (lead), project administration (lead), resources (equal), software (lead), validation (lead), visualization (lead), writing – original draft (lead), writing – review and editing (lead). **Atikul Islam Mithu:** investigation (equal), writing – review and editing (equal). **Auritro Sattar:** investigation (equal), writing – review and editing (equal). **Rasel Debbarma:** investigation (equal), writing – review and editing (equal). **Harish Debbarma:** investigation (equal), writing – review and editing (equal). **Sajid Hassan Prangon:** investigation (equal), writing – review and editing (equal). **Md. Ashiqun Nabi Litu:** investigation (equal), writing – review and editing (equal). **Safayat Hossain:** investigation (equal), writing – review and editing (equal). **Abdullah As Sadique:** investigation (equal), writing – review and editing (equal). **Fa‐Tu‐Zo Khaleque Mila:** investigation (equal), writing – review and editing (equal). **Sabit Hasan:** investigation (equal), writing – review and editing (equal). **Shimul Nath:** investigation (equal), project administration (supporting), writing – review and editing (equal). **Christian Roos:** conceptualization (supporting), funding acquisition (supporting), investigation (equal), methodology (supporting), resources (supporting), supervision (lead), validation (lead), writing – review and editing (equal).

## Conflicts of Interest

The authors declare no conflicts of interest.

## Supporting information


**File S1:** ece372279‐sup‐0001‐FileS1.docx.

## Data Availability

All data generated or analyzed during this study are included in this article and its File S1.
