# Supplementary material for: Improper Primate Release Fuels Interspecific Mating: Cases of Two Mixed‐Species Groups in Bangladesh
Source: Ecol Evol. 2025 Oct 16;15(10):e72279. doi: 10.1002/ece3.72279 (PMC12530873; doi:10.1002/ece3.72279)
Supplement: Supplementary file 1 — File S1: ece372279‐sup‐0001‐FileS1.docx. [file ECE3-15-e72279-s001.docx]

**Supplementary File**

**I.** Details of the mating events among a non-native male Assamese macaque and native female rhesus macaques in Satchari National Park, Northeast Bangladesh.

| **Events** | **Date** | **Time** | **Thrusting (second)** | **Place of mating** |
| --- | --- | --- | --- | --- |
| C1 | 11.11.2023 | 16:02 | 21 | Tree |
| C2 | 17.03.2024 | 12:36 | 7 | Tree |
| C3 | 21.03.2024 | 10:10 | 10 | Wall |
| C4 | 19.06.2024 | 11:15 | 6 | Ground |
|  |  | 11:17 | 4 | Ground |
|  |  | 11:20 | 11 | Ground |
| C5 | 16.07.2024 | 15:29 | 9 | Tree |
| C6 | 04.10.2024 | 14:10 | 15 | Ground |
| C7 | 07.02. 2025 | 09:03 | 13 | Ground |
| C8 | 17.03.2025 | 09:55 | 13 | Ground |
